# De novo mutations in MED13, a component of the Mediator complex, are associated with a novel neurodevelopmental disorder

**DOI:** 10.1007/s00439-018-1887-y

**Published:** 2018-05-08

**Authors:** Lot Snijders Blok, Susan M. Hiatt, Kevin M. Bowling, Jeremy W. Prokop, Krysta L. Engel, J. Nicholas Cochran, E. Martina Bebin, Emilia K. Bijlsma, Claudia A. L. Ruivenkamp, Paulien Terhal, Marleen E. H. Simon, Rosemarie Smith, Jane A. Hurst, The DDD study, Heather McLaughlin, Richard Person, Amy Crunk, Michael F. Wangler, Haley Streff, Joseph D. Symonds, Sameer M. Zuberi, Katherine S. Elliott, Victoria R. Sanders, Abigail Masunga, Robert J. Hopkin, Holly A. Dubbs, Xilma R. Ortiz-Gonzalez, Rolph Pfundt, Han G. Brunner, Simon E. Fisher, Tjitske Kleefstra, Gregory M. Cooper

**Affiliations:** 1 Human Genetics Department, Radboud University Medical Center, PO Box 9101, 6500 HB Nijmegen, The Netherlands; 2 Language and Genetics Department, Max Planck Institute for Psycholinguistics, Nijmegen, The Netherlands; 3 Donders Institute for Brain, Cognition and Behaviour, Nijmegen, The Netherlands; 4 HudsonAlpha Institute for Biotechnology, 601 Genome Way, Huntsville, AL 35806 USA; 5 University of Alabama at Birmingham, Birmingham, AL USA; 6 Department of Clinical Genetics, Leiden University Medical Center, Leiden, The Netherlands; 7 Department of Genetics, University Medical Center Utrecht, Utrecht University, Utrecht, The Netherlands; 8 Division of Genetics, Department of Pediatrics, Maine Medical Center, Portland, ME USA; 9 Great Ormond Street Hospital for Children, London, UK; 10 Wellcome Sanger Institute, Wellcome Genome Campus, Hinxton, Cambridge, CB10 1SA UK; 11 GeneDx, 207 Perry Parkway, Gaithersburg, MD 20877 USA; 12 Department of Molecular and Human Genetics, Baylor College of Medicine, Houston, TX USA; 13 Paediatric Neurosciences Research Group, University of Glasgow and Royal Hospital for Children, Glasgow, G51 4TF UK; 14 Wellcome Centre for Human Genetics, University of Oxford, Oxford, UK; 15 Ann and Robert H. Lurie Children’s Hospital of Chicago, Chicago, IL USA; 16 Division of Human Genetics, Cincinnati Children’s Hospital Medical Center, Cincinnati, OH USA; 17 Department of Pediatrics, College of Medicine, University of Cincinnati, Cincinnati, OH USA; 18 Division of Neurology, Children’s Hospital of Philadelphia, Philadelphia, PA USA; 19 Department of Clinical Genetics, GROW School for Oncology and Developmental Biology, Maastricht UMC, Maastricht, The Netherlands

## Abstract

Many genetic causes of developmental delay and/or intellectual disability (DD/ID) are extremely rare, and robust discovery of these requires both large-scale DNA sequencing and data sharing. Here we describe a GeneMatcher collaboration which led to a cohort of 13 affected individuals harboring protein-altering variants, 11 of which are de novo, in *MED13*; the only inherited variant was transmitted to an affected child from an affected mother. All patients had intellectual disability and/or developmental delays, including speech delays or disorders. Other features that were reported in two or more patients include autism spectrum disorder, attention deficit hyperactivity disorder, optic nerve abnormalities, Duane anomaly, hypotonia, mild congenital heart abnormalities, and dysmorphisms. Six affected individuals had mutations that are predicted to truncate the MED13 protein, six had missense mutations, and one had an in-frame-deletion of one amino acid. Out of the seven non-truncating mutations, six clustered in two specific locations of the MED13 protein: an N-terminal and C-terminal region. The four N-terminal clustering mutations affect two adjacent amino acids that are known to be involved in MED13 ubiquitination and degradation, p.Thr326 and p.Pro327. MED13 is a component of the CDK8-kinase module that can reversibly bind Mediator, a multi-protein complex that is required for Polymerase II transcription initiation. Mutations in several other genes encoding subunits of Mediator have been previously shown to associate with DD/ID, including *MED13L*, a paralog of *MED13*. Thus, our findings add *MED13* to the group of CDK8-kinase module-associated disease genes.

**Electronic supplementary material:**

The online version of this article (10.1007/s00439-018-1887-y) contains supplementary material, which is available to authorized users.

## Introduction

The introduction of next-generation sequencing techniques has rapidly improved the identification of genes that associate with rare disease. Although developmental delay (DD) and intellectual disability (ID) are relatively common (Boat and Wu 2015; Boyle et al. 2011), there is extreme genetic heterogeneity among affected patients and a large fraction of patients with DD/ID remain refractory to diagnosis (Vissers et al. 2016). In unsolved cases, the understanding of gene–disease relationships has greatly benefited from collaboration between clinical genetics teams (Sobreira et al. 2015). In fact, many recently discovered DD/ID genes have come from “matchmaking” (Au et al. 2015; Harms et al. 2017; Kernohan et al. 2017), where websites such as GeneMatcher (Sobreira et al. 2015) facilitate the comparison of patients with rare genotypes and phenotypes across the world.

Here we present the results of a collaboration facilitated by GeneMatcher (Sobreira et al. 2015) in which multiple clinical and research groups independently identified individuals with DD/ID and related phenotypes with rare protein-altering variation in *MED13*. This genotype-driven approach enabled us to characterize the phenotypes and mutational spectrum of a cohort of 13 patients, each with a likely pathogenic variant in *MED13*.

Although *MED13* has not been previously linked to a disorder, it is a paralog of *MED13L*, mutations of which have been found to cause ID, speech impairment and heart defects (Adegbola et al. 2015; Muncke et al. 2003; van Haelst et al. 2015). The gene products MED13 and MED13L are mutually exclusive components of the reversible CDK8-module of the Mediator complex, a multi-protein complex that is required for the expression of all protein-coding genes (Conaway et al. 2005; Malik and Roeder 2005). In this study, we show that variants in *MED13* are also associated with a neurodevelopmental disorder, and delineate the corresponding phenotypic features and mutational spectrum.

## Materials and methods

### Informed consent

Informed consent to publish de-identified data was obtained from all patients, either as part of the diagnostic workflow or as part of a research study (Bowling et al. 2017). Informed consent to publish clinical photographs was also obtained when applicable. Informed consent matched the local ethical guidelines.

### Exome/genome sequencing

In patients A, B, D, E, F, G, I, K, L and M, whole exome sequencing and variant filtering were performed as previously published (de Ligt et al. 2012; Deciphering Developmental Disorders 2015; Neveling et al. 2013; Sollis et al. 2017; Tanaka et al. 2015). In patient C, targeted Sanger sequencing was performed to confirm the presence of the MED13 variant (L131*) that was first identified in patient B. For patient H, whole genome sequencing was performed using Illumina’s HiSeq X ten platform. Sequencing reads were mapped against the hs37d5 reference using GATK. Variants were called using GATK’s Haplotype Caller. Variants were filtered using frequencies from the ExAC and gnomAD databases (mean allele frequency < 0.003) and for conservation using PhastCons (> 0.5) and PhyloP (> 4). For patient J, whole genome sequencing, variant prioritization, and Sanger validation were performed as previously described (Bowling et al. 2017). In each patient, the observed *MED13* mutation was considered to be the most likely contributor to the phenotype, and no additional pathogenic or likely pathogenic variants were found.

### Three-dimensional modeling

Protein modeling was performed as previously described (Prokop et al. 2017). Modeling of MED13 interacting with FBXW7 was performed using PDB 2OVQ, replacing molecule C with the MED13 amino acids 321–330. Binding energy was calculated following each patient variant insertion and energy minimization using AMBER14 force field (http://ambermd.org/) in YASARA.

### RNA isolation

2.5 mL of blood was collected in PAXgene RNA tubes (PreAnalytiX #762165) according to the manufacturer’s instructions and stored short-term at − 20 °C. RNA was isolated using a PAX gene Blood RNA Kit (Qiagen #762164) according to the manufacturer’s instructions. Isolated RNA was quantified by Qubit® (Thermo Fisher #Q32855).

### cDNA synthesis

First strand synthesis of cDNA was performed from 150 ng of RNA isolated from blood using Superscript™ III (Thermo Fisher #18080044) according to manufacturer’s instructions using random primers (Invitrogen #48190011) for +/− RT reactions. The products were diluted 1:10 in water before use in qPCR reactions.

### qPCR

qPCR was performed according to manufacturer’s protocols using Taqman gene expression master mix (ThermoFisher #4369016) and FAM-MGB Taqman probes directed against MED13 (ThermoFisher Hs01080701_m1 catalog #4331182) and GAPDH (ThermoFisher #4352934E). qPCR reactions were carried out in a QuantStudio 6 Flex Real-Time PCR system (Applied Biosystems) using 40 cycles of amplification. Raw C_T_ values were obtained, normalized first to the GAPDH loading control, and then to the proband. We tested an additional loading control [AGPAT-data not shown (ThermoFisher Hs00965850_g1; catalog #4331182)], but the data were like those normalized to GAPDH.

### Sanger sequencing

cDNA template was amplified using primers to the region of interest: 5′-CGAGGCTCTTATGGAACTGATGAATC-3′ (forward) and 5′-GATCCATCGTGCTTTCAGACACATC-3′ (reverse). No amplification was observed in the no RT condition. PCR conditions were: 500 nM primers, 3% DMSO, 1x Phusion HF (NEB #M0531L), 0.5 µL cDNA template, and cycling at (98 °C, 30 s), (98 °C, 10 s; 60 °C, 30 s; 72 °C, 45 s)x35, (72 °C, 7 m), (4 °C, ∞). The additional reverse primer 5′-AAATGCTTCATTGTTACCGTCAGCT-3′ and the additional forward primers 5′-TCCAAAAGAAACGATGTGAGTATGCAG-3′, 5′-CTCTCTTCAGCCAGTTCTTCAGGAT-3′, 5′-ACAATTTCATAAAATGGCTGGCCGA-3′, 5′-CGAGGCTCTTATGGAACTGATGAATC-3′, 5′-GTGCTTTCTCCATTTGCTCTTCCTT-3′ were used for sequencing, along with the primers used for amplification from cDNA. Chromatograms were quantified using ab1PeakReporter (Thermo Fisher).

### Western Blot

Whole blood was collected using cell processing tubes (BD #362760), isolated according to the manufacturer’s instructions, and stored in liquid nitrogen in CTS™ Synth-a-Freeze^®^ Medium (Thermo Fisher # A13713-01) until use. As a control for antibody specificity, MED13 was knocked down in neural precursor cells (clone BC1, MTI-GlobalStem #GSC-4311) by generating stable lines using puromycin selection expressing shRNA against MED13 (Sigma Aldrich # SHCLNG-NM_005121; TRCN0000234904) compared to a GFP shRNA control in the same vector (Addgene # 30323). Cell pellets were processed using the NE-PER™ (Thermo Fisher #78833) nuclear and cytoplasmic extraction kit according to the manufacturer’s instructions, and nuclear extracts were used for the blot shown (whole cell extracts, even at very high concentrations, did not produce sufficient signal). 60 µg of protein was loaded for patient blood samples, and 15 µg of protein was loaded for neural precursor cell samples. Blots were blocked for 1 h at room temperature in LICOR blocking buffer (LICOR #927-40000), then blots were probed (with washes in PBS-T (0.05% Tween-20) and a secondary probe for 1 h after each primary probe) with 1:250 rabbit anti-MED13 (Bethyl #A301-277A) for 3 days at 4 °C, 1:1,000 mouse anti-HDAC2 (clone 3F3, SCBT #sc-81599) overnight at 4 °C as a loading control, and 1:1000 rabbit anti-HSP90 (abcam #ab115660) overnight at 4 °C as an additional loading control. Secondary probes were used at 1:20,000 (LICOR #926-32211 and #926-68070). Three other primary antibodies were tested for MED13, but did not show sufficient signal to detect MED13 in blood despite detecting MED13 in neural precursor nuclear lysates: Bethyl #278A, Abcam #ab49468, and Abcam #ab76923 (data not shown).

### Statistical enrichment of *MED13* variants in DD/ID cohorts

We compared the frequency of observed de novo MED13 variation identified in two large sequencing cohorts to the expected frequency of variation in *MED13* based on its gene specific mutation rate (Samocha et al. 2014) using an Exact Poisson Test in R (R Core Team. R: A language and environment for statistical computing (http://www.r-project.org). Vienna).

## Results

### Phenotypes

We collected detailed clinical information of 13 patients with rare, protein-altering *MED13* variants. Eleven variants were confirmed to be de novo, and one patient (patient B) inherited the variant from her mother who is also affected (patient C). Phenotypic data summarizing the spectrum of features of this cohort of 13 patients are shown in Table 1.

**Table 1:** Clinical features of patients with *MED13* mutations and molecular characterization

	Patient A	Patient B	Patient C	Patient D	Patient E	Patient F	Patient G	Patient H	Patient I	Patient J	Patient K	Patient L	Patient M
Molecular characterization													
cDNA variant (NM_005121.2)	c.125del	c.392T>G	c.392T>G	c.977C>T	c.975_977delTAC	c.979C>T	c.980C>A	c.1618C>A	c.1745T>A	c.4198C>T	c.4487delC	c.6178C>A	c.6191C>T
Predicted protein effect	P42Lfs*6	L131*	L131*	T326I	T326del	P327S	P327Q	P540T	L582*	R1400*	T1496Mfs	Q2060K	A2064V
CADD v.1.3	31.0	37.0	37.0	25.0	20.5	23.4	25.2	26.3	40	41	35	24.1	25.7
GERP + + RS	5.67	5.32	5.32	5.5	5.5	5.5	5.5	6.16	6.02	4.58	5.86	6.04	6.04
Inheritance	De novo	Maternal (daughter of pt. C)	Unknown	De novo	De novo	De novo	De novo	De novo	De novo	De novo	De novo	De novo	De novo
Clinical characterization													
Gender	M	F	F	M	F	M	F	M	M	F	M	F	F
Age at last visit (years)	8	5	32	19	9	11	3	10	6	13	5	10	6
Height	+ 0.6 SD	+ 0.5 SD	average	− 0.2 SD	+ 2.2 SD	+ 0.5 SD	+ 0.3 SD	− 0.9 SD	0 SD	− 0.7 SD	− 2 SD	+ 0.5 SD	− 2 SD
Weight (for height)	+ 1.8 SD	+ 1.9 SD	average	− 0.8 SD	+ 1.2 SD	− 0.9 SD	− 1.1 SD	+ 0.6 SD	+ 0.5 SD	0 SD	− 1.6 SD	+ 0.4 SD	+ 0.7 SD
Head circumference	− 1 SD	+ 0.2 SD	NA	− 0.5 SD	+ 1.1 SD	− 0.9 SD	− 0.3 SD	− 1.5 SD	− 0.5 SD	NA	0 SD	− 2 SD	+ 1 SD
Intellectual Disability (ID) / Developmental Delay (DD)	Mild ID	Mild/borderline ID	Borderline ID (IQ 80–85)	Mild ID (IQ 65)	Mild ID	Mild ID	DD	Borderline ID (IQ 85 with working memory score 68 on WISC-IV)	Mild ID (IQ 61)	Moderate ID	DD	DD	Mild/borderline ID (IQ 70)
Speech delay/disorder	+ Speech apraxia with mixed receptive and expressive language disorder, limited verbal expression and language-based learning disorder	+ Delayed speech development, mild articulation problems	+ Delayed speech development, expressive language problems in childhood. At adult age only sporadic and mild word-finding problems	+ Delayed speech development, mild articulation problems, normal language comprehension	+ Moderate mixed receptive and expressive language disorder, decreased vocabulary and language formulation difficulties	+ Delayed expressive and receptive language	+ Mainly expressive speech problems	+/− Borderline (verbal comprehension score = 87 on WISC-IV)	+ At age 6y expressive and receptive speech at age equivalent < 2y	+ Moderate expressive language disorder	+ Severe speech disorder with regression, speech apraxia, receptive language is fine	+ Severe speech delay, 5–10 words	+ Severe speech/language disorder, expressive language most affected, signs of speech apraxia
Delayed motor development	+ (Only fine motor skills delayed)	− (walked at 14 m)	− (walked on time)	+ (walked at 20 m)	+ (walked at 25 m)	+ (walked at 22 m)	+ (walked at 26 m)	− (walked before 12 m)	+ (walked at 2 years; early delays, now mostly on target with peers)	NR	− (walked at 12 m)	−	+ (walked at 20 m)
Autism spectrum disorder (ASD) /ADHD	ADHD	NA	−	−	−	ASD	ASD	−	−	ASD, ADHD	ASD	ASD, ADHD	−
Brain MRI	Normal	NA	NA	Normal	Bulbous splenium of corpus callosum (likely normal variant)	Normal	Normal	Small area of abnormal signal in left occipital lobe	Normal	NA	NA	Normal	Mild frontal atrophy, otherwise normal
Eye/vision abnormalities	Astigmatism	probably amblyopia	−	Visual impairment, pale optic nerves	Congenital nystagmus, outer retinal atrophy temporal to both optic discs, optic nerves low normal in size on MRI	−	Strabismus, papil edema	−	Astigmatism	NR	NR	Duane anomaly	Duane anomaly
Heart abnormalities	NR	−	NR	−	History of murmur, normal echo and ECG	Dilated aortic root and pulmonary artery	−	NR	NR	Subaortic stenosis	NR	NR	NR
Chronic obstipation	NR	NR	+	NR	+	NR	+	NR	NR	NR	NR	NR	+
Other features (features reported in two unrelated patients in bold)		Sloping shoulders, small and laterally deviated halluces	Small and laterally deviated halluces	Kyphosis, pes cavus	Hypotonia, mild proximal weakness, fatigues easily, clumsy gait, transient lactic acidosis with illness, congenital left hip dysplasia	Hypotonia, Conductive hearing loss, Mild scoliosis, pes cavus	Hypotonia	Epilepsy (drug-resistant with myoclonic-atonic seizures)		Chronic sleep disturbances	Chronic sleep disturbances	Conductive hearing loss, Precocious puberty	

*NR* not reported, *NA* not assessed

All patients had developmental delays with varying severity and course. In the patients that underwent formal intelligence testing, total IQ levels varied from 85 (lower range of normal IQ) to an IQ between 35 and 50 (moderate ID). Five patients had an Autism Spectrum Disorder (ASD), and three patients were diagnosed with Attention Deficit Hyperactivity Disorder (ADHD). All patients had speech delays and/or disorders, with delayed milestones in speech and language development. While several patients had expressive and receptive language problems, in the majority of patients, speech production was significantly more impaired than language comprehension. Three patients (patient A, K and M) showed characteristics of speech apraxia, a developmental speech disorder in which affected individuals have difficulties accurately programming the motor sequences required to produce fluent speech. Patient A had a mild ID, but showed speech apraxia with a mixed receptive and expressive language disorder, and limited verbal expression at the age of 8 years. Patient M had a non-verbal IQ of 70 with a severe speech/language disorder. Her expressive speech was severely affected, with signs of speech apraxia. At the age of 8 years she only used single words and very short sentences. Patient K developed some speech capabilities, but showed regression at the age of 13 months and has since remained non-verbal.

Seven of 13 patients showed delays in motor development, most of which affected at least the gross motor skills (6 of 7), although one patient was reported to have only fine motor delays. Three patients had hypotonia (patient E, F and G). One patient (patient H) developed severe drug-resistant myoclonic-atonic epilepsy at 4 years of age with generalized clonic, myoclonic, atonic, tonic and atypical absence seizures. MRI screening of this patient showed a small abnormality in the left occipital lobe of his brain that did not correspond to the electrophysiological onset or the semiology of his seizures. In other patients, MRI scans were not performed or showed no clear abnormalities, except for mild frontal atrophy in patient M.

Eight patients (62%) presented with eye or vision abnormalities. Two patients (patients L and M) presented with Duane anomaly, a congenital type of strabismus that is characterized by non-progressive horizontal ophtalmoplegia and retraction of the globe with attempted adduction, together with narrowing of the palpebral fissure (Andrews et al. 1993). One patient (patient G) had strabismus, two patients had astigmatism (patient A and I), and one patient (patient E) had congenital nystagmus. While only one patient (patient D) had a visual impairment, three patients had optic nerve abnormalities: pale optic nerves in patient D, papilledema in patient G, and in patient E outer retinal atrophy temporal to both optic discs was reported with relatively small optic nerves on a MRI-scan.

Several other interesting phenotypes were observed in at least two patients in the cohort. Four patients presented with chronic obstipation (patients C, E, G and M). Two patients had conductive hearing loss (patients F and L). Two patients had congenital heart abnormalities: a mildly dilated aortic root and pulmonary artery (both improving over time) in patient F, and a subaortic stenosis in patient J. Two patients were reported to have chronic sleep issues (patient J and K).

**Fig. 1:**
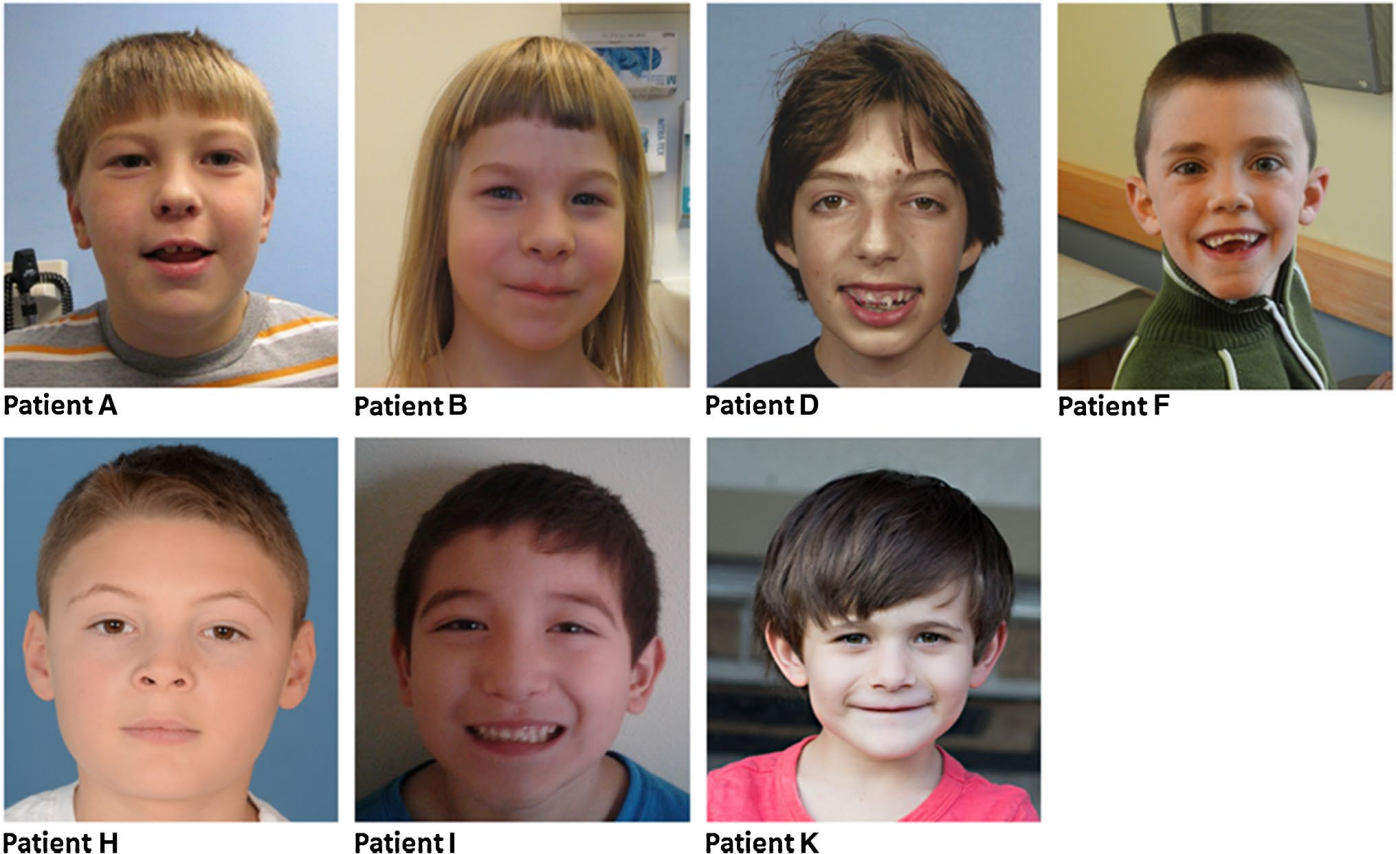
Facial phenotypes of seven individuals with a *MED13* variant. Overlapping facial characteristics include peri-orbital fullness, narrow palpebral fissures, a broad and high nasal bridge, full nasal tip, synophrys, flat philtrum, wide mouth and a thin upper lip

Overlapping facial characteristics were reported, including widely spaced eyes with narrow palpebral fissures and peri-orbital fullness, a broad and high nasal bridge, full nasal tip, synophrys, a flat philtrum and a wide mouth with thin upper lip (Fig. 1).

### Variants and predicted consequences

**Fig. 2:**
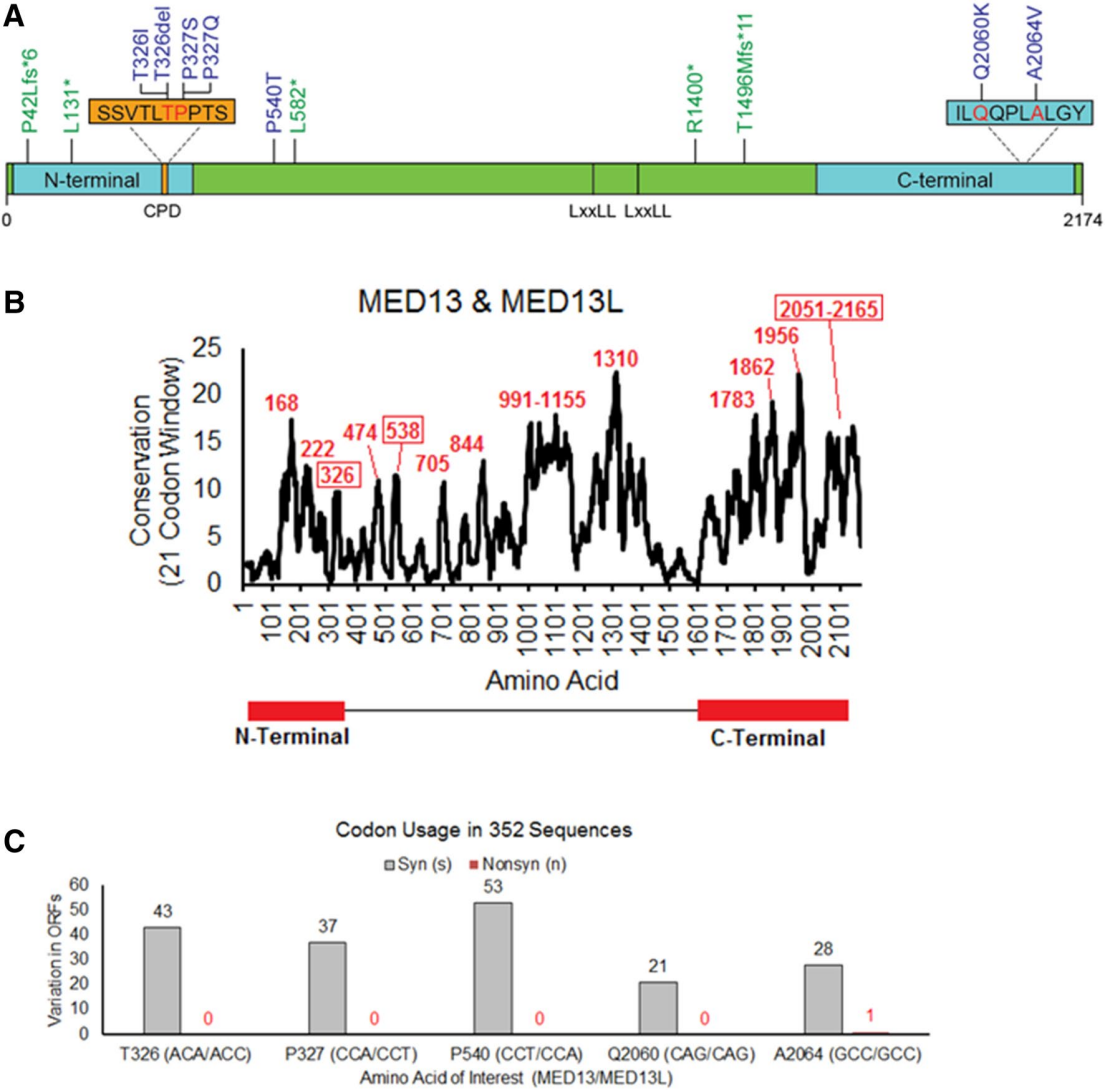
Analysis of mutations: location, conservation and codon usage of variant sites. **a** Identified mutations are shown within a linear representation of the MED13 protein, consisting of 2174 amino acids. Missense mutations and the in-frame deletion are shown in blue, and nonsense and frameshift mutations in green. Six of the seven non-truncating mutations in our MED13 cohort cluster in two small regions within the N-terminal and C-terminal domains of the MED13 protein. Affected amino acids p.Thr326 and p.Pro327 and are part of a conserved phosphodegron (CPD), which is shown in orange. Two LxxLL nuclear receptor-binding motifs are also noted. **b** Analysis of conservation throughout the protein was performed using amino acid selection scores as previously published (Prokop et al. 2017), using a 21 codon sliding window for both MED13 and MED13L aligned such that the most selected motifs of a protein are identified as peaks. The center of each highly conserved linear motif is labeled and those containing variants described in this paper are boxed. **c** Codon usage throughout evolution for the locations of all missense mutations and the in-frame deletion. All five sites are under high selection with multiple synonymous (Syn, gray) amino acids in 352 open reading frames (ORFs) of MED13 and MED13L with only a single nonsynonymous (Nonsyn, red) change. Numbers indicate instances where ORFs in other species deviate from the conserved codon usage. Of note, for three locations (326, 327 and 540) the codon used differs between MED13 and MED13L with the amino acid conserved. In these cases, numbers indicate where ORFs in other species deviate from conserved codon usage in their respective ortholog

The *MED13* transcript (NM_005121.2) encodes a large protein consisting of 2174 amino acids (NP_005112.2). The Pfam database characterizes two domains within the MED13 protein: an N-terminal domain (aa 11–383) and a C-terminal domain (aa 1640–2163), as shown in Fig. 2a. Analysis of conservation across the length of the protein indicates several highly conserved residues that lie between these two domains (Fig. 2b).

**Fig. 3:**
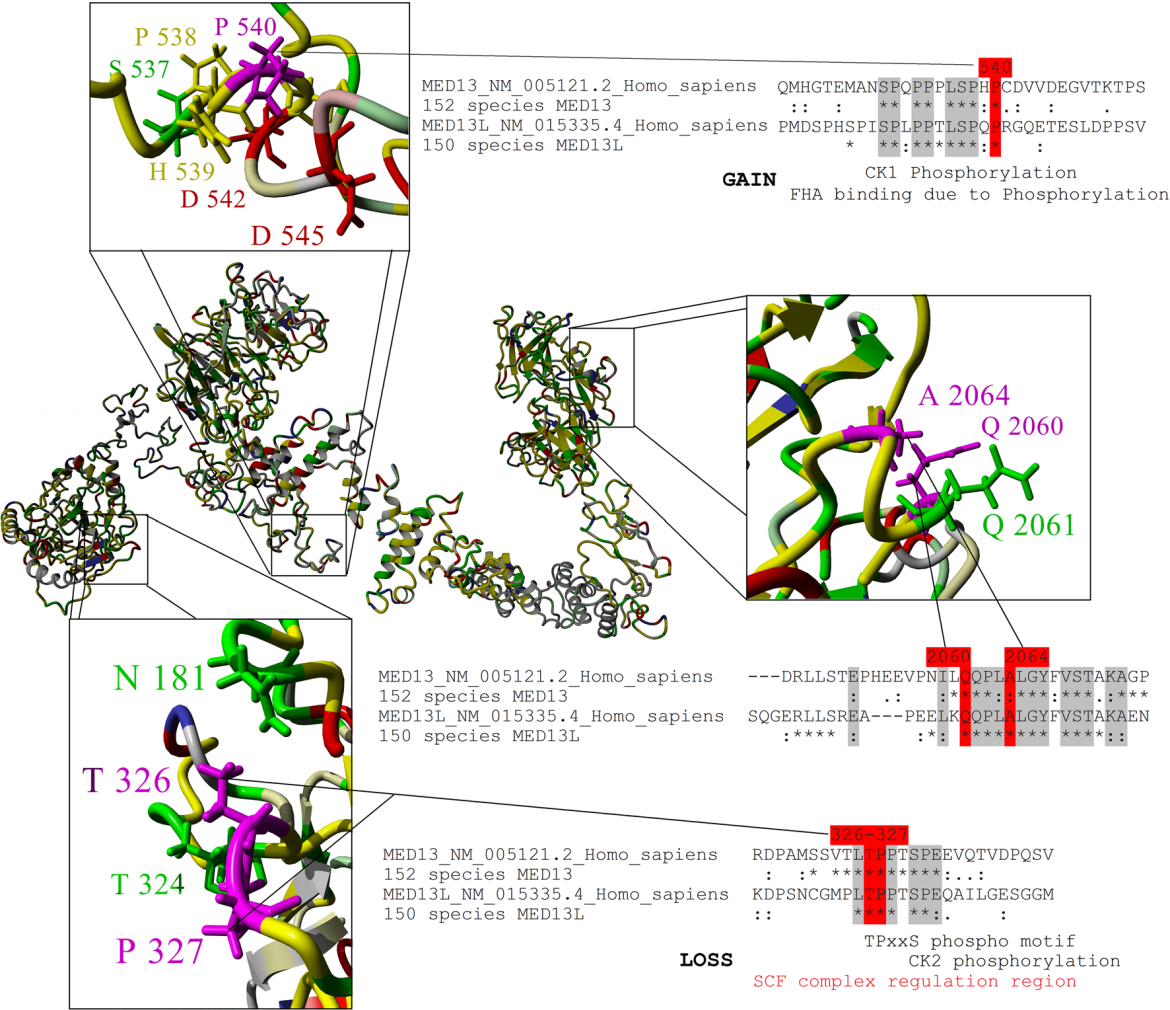
Location of missense mutations and in-frame deletion in three-dimensional structure of MED13 and conservation of affected amino acids. A full model of MED13 protein created with I-TASSER modeling was combined with 152 species sequences for MED13 using ConSurf mapping. Amino acid coloring is as followed: gray = not conserved, yellow = conserved hydrophobic, green = conserved hydrophilic, red = conserved polar acidic, blue = conserved polar basic, magenta = conserved human variants of interest. A zoomed in view of the three different affected regions are shown, along with amino acid alignments from MED13 and MED13L. An asterisk (*) indicates 100% conservation in all sequences and a colon (:) indicates functional conservation. Linear motifs mapped with the Eukaryotic Linear Motif tool are shown below sites for 326–327 and 540

All 12 unique variants found in our patients are absent from the gnomAD database (Lek et al. 2016) and TOPMED Bravo database (https://bravo.sph.umich.edu/freeze3a/hg19/) and are predicted to be highly deleterious by CADD v1.3 (Kircher et al. 2014), with scores ranging from 20.5 to 41 (Table 1). Six patients had five unique variants that are predicted to be truncating: three nonsense mutations (p.Leu131* in Patients B and C, p.Leu582* in Patient I and p.Arg1400* in Patient J) and two frameshift variants leading to a premature stop codon (p.Pro42Leufs*6 in patient A and p.Thr1496Metfs*11 in Patient K). The remaining variants include six missense variants and a single amino acid deletion. These seven variants form two apparent clusters: one in the N-terminal conserved phosphodegron domain and the other in the C-terminal domain (Fig. 2a). These seven variants were all found to lie within motifs that are highly conserved between MED13 and MED13L (Fig. 2b) and affect sites under high codon selection (Fig. 2c). These missense variants and the in-frame deletion are each located on surface-exposed sites within a three-dimensional model of the MED13 protein (Fig. 3). The four mutations that cluster in the N-terminal domain affect two adjacent amino acids (p.Thr326 and p.Pro327) that are known to be part of a conserved phosphodegron that is required for binding with SCF-Fbw7 ubiquitin ligase for degradation (Davis et al. 2013). Using interaction data from Davis et al. and PDB structure 2OVQ, which has Fbw7 interacting with a similar motif as MED13, we modeled this interaction for MED13 followed by insertion of each variant and calculation of binding energy. All four variants (p.Thr326Ile, p.Thr326del, p.Pro327Ser, p.Pro327Gln) are predicted to alter the phosphorylation and Fbw7 interaction with drastic decreases in binding energy to Fbw7 (Supplementary Fig. 1). The two missense changes clustering in the C-terminal portion of the protein (p.Gln2060Lys and p.Ala2064Val; in patients L and M, respectively) were also studied in more detail. One of the changes (p.Ala2064Val) is predicted to be structure-altering through increasing hydrophobic collapse, secondary structure formation, and increasing aliphatic index of a surface exposed linear motif. This results in a decrease of the regions linear interacting peptide potential that is highly conserved and likely functional (Supplementary Fig. 2). The remaining missense variant (p.Pro540Thr in Patient H) lies within a highly conserved linear motif centered near amino acid 538 (Fig. 2b); it results in the formation of a high probability Casein Kinase 1 phosphorylation motif, which could lead to additional interaction with proteins containing forkhead-associated domains when analyzed through the ELM database (Dinkel et al. 2016) (Fig. 3).

### Effects of truncating *MED13* mutation on transcript and protein levels

**Fig. 4:**
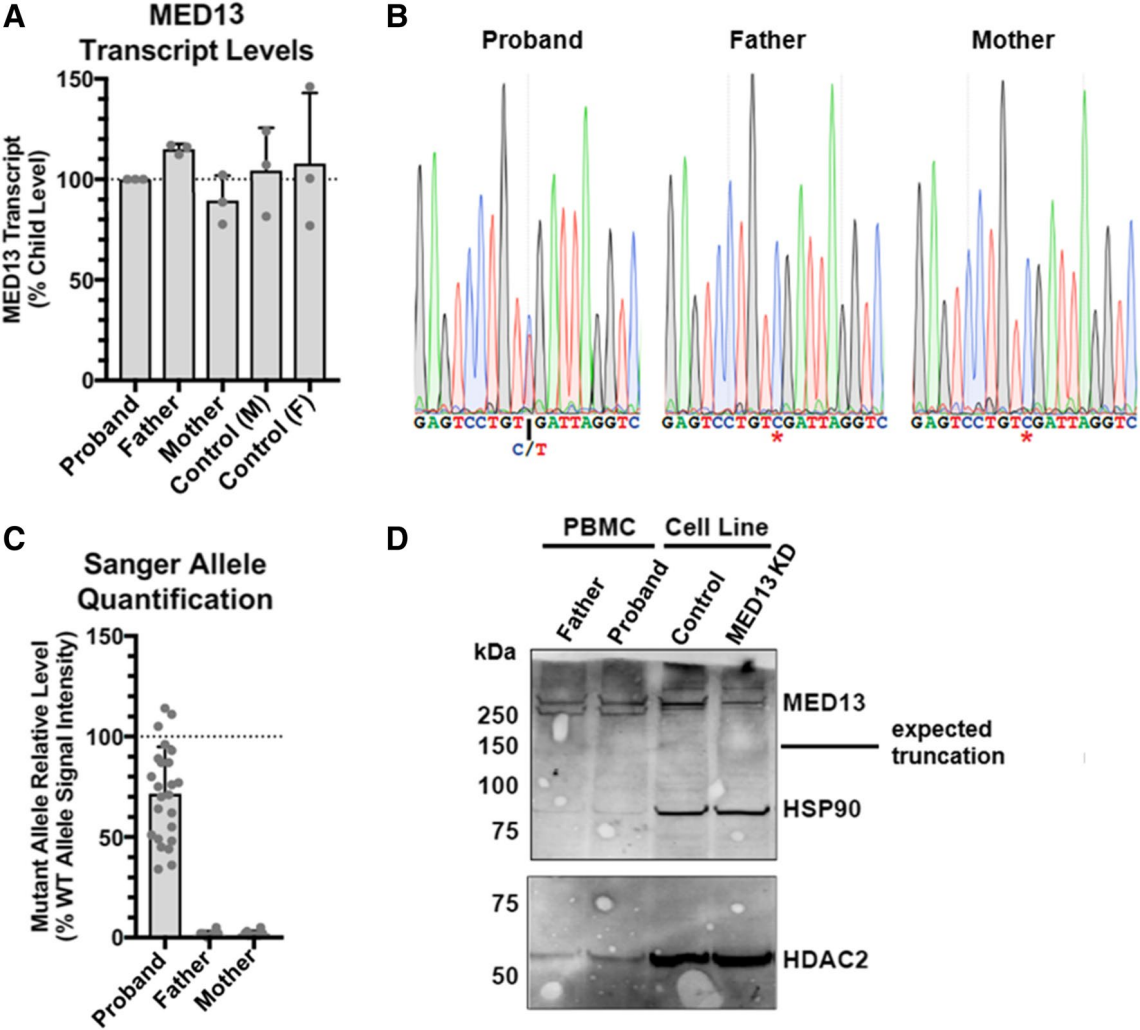
Analysis of transcript and protein levels in patient with nonsense mutation. **a** Level of MED13 transcript was measured by qPCR and normalized to GAPDH and proband (patient J). No differences were detectable between groups (One-way ANOVA *p* = 0.5913). An additional loading control (AGPAT) produced very similar results (data not shown). **b** Representative Sanger traces from cDNA amplicons demonstrating the presence of the variant in the proband, and absence in the father and mother. **c** Quantification of the chromatograms of all Sanger sequences reveals less signal from the base on the mutant allele (*p* < 0.0001 by paired t-test compared to the wildtype base signal by trace). The father and mother do not have any signal at the mutant base above the level of noise. **d** Western blot for MED13 (and HSP90 and HDAC2 as loading controls) from nuclear extracts of patient peripheral blood mononuclear cells or a neural precursor cell line (present to demonstrate antibody specificity with a knockdown (KD) control). If the nonsense mutation resulted in a stable protein, a product at approximately 150 kDa would be expected, which is not present. No protein was recoverable from the blood sample from the mother

As truncating mutations often lead to nonsense-mediated decay and haploinsufficiency, we aimed to examine the effects of a truncating *MED13* mutation on levels of *MED13* transcript and MED13 protein. We performed RT-PCR on cDNA transcribed from RNA of patient J, who was heterozygous for a nonsense mutation (c.4198C > T; p.Arg1400*). We compared the *MED13* transcript level of the patient to her biological parents and two healthy controls (Fig. 4a). No differences in *MED13* transcript levels were detectable between the affected patient and the unaffected parents or controls (One-way ANOVA *p* = 0.5913). Sanger sequencing of cDNA amplicons from the child demonstrated the presence of the aberrant transcript in the child (Fig. 4b), at ~ 70% levels relative to the normal transcript (Fig. 4c). To assess the effect of the nonsense mutation on protein levels, a western blot was performed on nuclear extracts from mononuclear blood cells of the patient and controls (Fig. 4d). While full-length MED13 protein was present in the patient (and in the controls), no truncated MED13 protein product could be detected. The MED13 protein level of the patient was not clearly different compared with the MED13 protein level of the father.

### Enrichment of de novo *MED13* variants in DD/ID cohorts

We quantified the extent of enrichment of de novo variants in *MED13* within DD/ID-affected probands. We used only the two largest cohorts considered within this study, each of which yielded at least two de novo *MED13* variants. Five patients described here (A, E, F, I, and K) come from a cohort of 11,149 affected individuals, and two patients, one of which is described here (patient L), were identified within the Deciphering Developmental Disorders (DDD) study of 4293 trios (Deciphering Developmental Disorders 2017). Both studies suggest a rate close to 1 de novo variant affecting *MED13* per ~ 2200 DD/ID-affected individuals. When comparing the number of observed de novo mutations in *MED13* to the expected number based on the gene specific mutation rate of *MED13* for missense, splice-site, nonsense and frameshift mutations [6.237 × 10^−5^ per chromosome (Samocha et al. 2014)], we find evidence for a significant enrichment among DD/ID-affected individuals (7 variants in 30,884 alleles; *p* = 0.00371).

## Discussion

By molecular and clinical characterization of a cohort of 13 patients with variants in *MED13*, we here provide evidence for a new neurodevelopmental disorder. This *MED13*-associated syndrome is characterized by DD/ID with speech delay and/or speech disorders. Additionally a broad spectrum of other common features is seen, including ASD, ADHD, various eye abnormalities and mild facial dysmorphisms. Based on the phenotypes of patients presented here, we do not yet see a clear genotype-phenotype correlation between type and location of the mutations and severity of clinical features. However, it is notable that the two patients with Duane anomaly have a missense mutation in a similar location in the C-terminal domain of the MED13 protein, and that the optic nerve abnormalities are reported in patients with mutations affecting residues p.Thr326 or p.Pro327 only.

MED13 is a component of the CDK8-kinase module, which can reversibly bind the Mediator complex. Mediator is a multi-protein complex that is required for assembly and stabilization of the pre-initiation complex, which is essential for transcription initiation (Chen et al. 2012; Hantsche and Cramer 2017). The core function of Mediator is to transmit signals from various transcription factors to RNA polymerase II (Pol II) (Allen and Taatjes 2015). Binding of the CDK8-module to Mediator has been reported to prevent the association of Mediator with the Pol II pre-initiation complex, thus preventing transcription initiation and/or re-initiation. In this way, the CDK8-module is considered a key molecular switch in Pol II mediated transcription (Knuesel et al. 2009). MED13, as well as the other subunits of the CDK8-module, are known to be critical regulators of developmental gene expression programs in Drosophila, zebrafish and *C. elegans* (Carrera et al. 2008; Poss et al. 2013). MED13, or its paralog MED13L, forms a direct connection of the CDK8 module with the core Mediator complex (Daniels 2013), and protein turnover of MED13 (or MED13L) may be critical in modulating the pools of Mediator-CDK8 kinase complex in cells (Davis et al. 2013; Knuesel et al. 2009; Tsai et al. 2013).

Three missense mutations (p.Thr326Ile, p.Pro327Ser and p.Pro327Gln) and one in-frame-deletion (p.Thr326del) in our cohort are likely to affect MED13 protein turnover due to their location within a conserved phosphodegron. This phosphodegron is recognized by the SCF-Fbw7 ubiquitin ligase, which targets the MED13 protein for ubiquitination and degradation (Davis et al. 2013). In fact, it has already been shown that a specific amino acid substitution at position 326 in MED13 (p.Thr326Ala) leads to impaired binding of Fbw7 to the phosphodegron of MED13/MED13L, thus preventing MED13/MED13L ubiquitination and degradation (Davis et al. 2013). Therefore, a variant at this position may lead to increased levels of MED13 protein in the cell. As Fbw7 is proposed to target only MED13 or MED13L proteins that are bound to the core Mediator complex (Davis et al. 2013), these mutations may have an effect on the CDK8 module-Mediator association and subsequently on transcription regulation. The potential effects of the p.Pro540Thr missense variant are also intriguing. Protein modeling suggests that this variant could introduce an additional Casein Kinase 1 phosphorylation site, thus potentially increasing interactions with forkhead-associated domains involved in protein–protein interactions.

We also observed five unique mutations predicted to truncate MED13. In assessments of RNA and protein levels in Patient J and her unaffected parents, the variant transcript was detected in the proband but no truncated protein could be observed. While these results are inconclusive with regards to the molecular mechanism of pathogenicity in this particular proband, loss-of-function mechanisms remain an attractive possibility. Patterns of variation in *MED13* in human population databases indicate that *MED13* is relatively intolerant to loss-of-function variation; MED13 has a Rare Variant Intolerance Score (RVIS) that ranks among the top 1.66% of all genes (Petrovski et al. 2013) and an ExAC pLI score of 1.00 (Lek et al. 2016).

We show an enrichment of de novo *MED13* mutations compared to what is expected under a null model (*p* = 0.00371) in two large ID/DD patient cohorts. We acknowledge that this *p* value does not exceed a genome-wide evidence threshold and by itself proves association. However, the enrichment *p* value does not account for five de novo variants described here from smaller cohorts that were discovered independent of, and prior to, assessment of the statistical evidence from the larger cohorts. We also observed clustering of missense mutations in our cohort, which by itself is an argument for pathogenicity (Lelieveld et al. 2017). Additionally, independent genetic studies also support the disease relevance of variation in *MED13*. There is one report of an 800-kb microdeletion including *MED13* and five other genes in a patient with moderate ID, short stature, mild dysmorphisms, and hearing loss (Boutry-Kryza et al. 2012); the authors proposed *MED13* as the most likely causal candidate gene. Additionally, a de novo frameshift (p.Pro286Leufs*86) and a de novo variant that likely affects splicing (D + 3; c.814+3A>G) were observed in a cohort of 2508 probands with ASD (Iossifov et al. 2014), and three rare protein-altering variants in *MED13* (p.Ala418Thr, p.Arg512*, p.Tyr1649*) were also found in a separate ASD cohort (Yuen et al. 2017).

Other Mediator subunits, including other CDK8-kinase module-associated disease genes, have been associated with various neurodevelopmental disorders. Variants in *MED12* have been associated with ID syndromes with congenital abnormalities, including Opitz-Kaveggia syndrome (MIM 305,450) (Risheg et al. 2007), Lujan-Fryns syndrome (MIM 309,520) (Schwartz et al. 2007) and X-linked Ohdo syndrome (MIM 300,896) (Vulto-van Silfhout et al. 2013). Mutations in *MED12* have also been associated with intellectual disability. In addition to ID and speech delays both MED12 patients and several MED13 probands described here present with eye abnormalities (eye movement disorders, and abnormalities of the retina and optic nerves) (Clark et al. 2009; Donnio et al. 2017) and chronic obstipation (Donnio et al. 2017; Lyons 1993). In addition to the MED12 subunit, a disruption of *CDK19* was reported in a patient with ID, microcephaly and congenital retinal folds (Mukhopadhyay et al. 2010).

It is of particular relevance to this study that variation in the *MED13-*paralog *MED13L* has been shown to cause a neurodevelopmental disorder as well (Asadollahi et al. 2013). Given the similar molecular roles for MED13 and MED13L, we aimed to compare and contrast phenotypes presented by both groups of individuals using information provided in the literature. The main phenotypic characteristics of MED13L-associated syndrome are (borderline) ID with delayed speech and language development, and a variable spectrum of other features including autism, hypotonia, characteristic facial features and heart defects (Adegbola et al. 2015; Caro-Llopis et al. 2016; Martinez et al. 2017; Muncke et al. 2003; van Haelst et al. 2015). Many of these features clearly overlap with the phenotypes in our *MED13* cohort. However, similar to the heterogeneity observed here in patients with *MED13* variation, the spectrum of phenotypes observed among *MED13L* mutation carriers is quite broad. The identification and detailed phenotyping of additional patients with *MED13* and *MED13L* mutations is needed to elucidate the complete spectrum of associated features, and to reveal the similarities and differences between the two syndromes.

We believe that the data presented in this study coupled to the additional evidence available from other studies strongly support the conclusion that rare protein-altering variation in *MED13* underlie a new neurodevelopmental disorder. Key results from this study include: a significant enrichment of de novo mutations in *MED13* within ID/DD cohorts (*p* = 0.00371); the clustering and conservation levels of the positions affected by the observed missense variation (Fig. 2a, b); the computationally predicted deleteriousness of the observed mutations (Table 1; Fig. 3, Supplementary Fig. 1); and the overlap of phenotypic features among the 13 patients presented here, including speech difficulties (13/13), intellectual disability (at least 9/13), and eye or vision problems (8/13). Supporting evidence from other studies include: the existence of mutations affecting *MED13* in at least six independent families affected by pediatric neurodevelopmental disorders; the intolerance of *MED13* to mutations in the general human population (pLI = 1.00, RVIS score of 1.66%); and the previously established disease-associations of several other Mediator subunits, including *MED13L*, a functionally related paralog of *MED13*. While the precise pathogenic mechanisms have yet to be elucidated—some of the mutations observed here are predicted to stabilize MED13 protein while others are predicted to lead to loss-of-function—we find it highly likely that mutational disruption of normal MED13 function leads to disease, adding *MED13* to the list of Mediator-associated, in particular CDK8-kinase module-associated, neurodevelopmental disorders.

## Electronic supplementary material

Below is the link to the electronic supplementary material.

**Supplementary** Figure 1: Analysis of variants at position 326-327 in relation to Fbw7-interaction. The interaction of MED13 with Fbw7 was modeled, by using PDB structure 2OVQ and amino acids 321-330 of the MED13 protein. All four different variants in our cohort that affect this binding region (T326I, T326del, P327S, P327Q) were subsequently inserted in the model, and binding energy was calculated using AMBER14 force field (http://ambermd.org/) in YASARA. All four variants are predicted to alter the phosphorylation and Fbw7 interaction with a severe decrease in binding energy to Fbw7 (PNG 2012 KB)

**Supplementary Figure 2** Structural packing of MED13 and variants at amino acid position 2060-2064. A) MobiDB breakdown (http://mobidb.bio.unipd.it/Q9UHV7/predictions) for MED13 showing structural disorder (orange) from various databases, linear interacting peptides (LIPs, green) helical prediction (pink), beta sheet prediction (light orange), and rigidity (magenta). The 2060-2064 region is boxed in red with low prediction of disorder and a predicted LIP from 2060-2070, suggesting this highly conserved surface exposed region has a high potential to form secondary structure when bound to some unknown protein binding partner. B) Jpred4 secondary structure predictions(Drozdetskiy et al. 2015) showing predicted changes in secondary structures, with a score of 9 being most likely to form secondary structure at each residue. p.Ala2064Val has highest probability to form stable secondary structure (average residue score of 7.05 compared to the wild type WT 5.6 and p.Gln2060Lys of 5.7). A variant increasing secondary structure would decrease formation rates with the unknown binding partner, thus likely resulting in loss of binding. C) Aliphatic index score(Ikai 1980) showing p.Ala2064Val to increase thermostability of the linear motif. An increase in intrinsic thermostability likely decreases formation rates with the unknown binding partner similar to secondary structure predictions. D) Location and effect of the two missense mutations p.Gln2060Lys and p.Ala2064Val shown on our predicted models for the region (PNG 351 KB)

## References

[CR1] Adegbola A (2015). Redefining the MED13L syndrome Eur. J Hum Genet.

[CR2] Allen BL, Taatjes DJ (2015). sThe Mediator complex: a central integrator of transcription. Nat Rev Mol Cell Biol.

[CR3] Andrews CV, Hunter DG, Engle EC (1993) Duane Syndrome. In: Pagon RA et al (eds) GeneReviews(R). Seattle (WA) 20301369

[CR4] Asadollahi R (2013). Dosage changes of MED13L further delineate its role in congenital heart defects and intellectual disability. Eur J Hum Genet.

[CR5] Au PYB (2015). GeneMatcher aids in the identification of a new malformation syndrome with intellectual disability, unique facial dysmorphisms, and skeletal and connective tissue abnormalities caused by de novo variants in. HNRNPK Hum Mut.

[CR6] Boat TF, Wu JT (eds) (2015) mental disorders and disabilities among low-income children. Washington (DC). 10.17226/21780 26632628

[CR7] Boutry-Kryza N (2012). An 800 kb deletion at 17q23.2 including the MED13 (THRAP1) gene, revealed by aCGH in a patient with a SMC 17. Am J Med Genet A.

[CR8] Bowling KM et al (2017) Genomic diagnosis for children with intellectual disability and/or developmental delay. Genome Med 9:43. 10.1186/s13073-017-0433-1 10.1186/s13073-017-0433-1 PMC5448144 28554332

[CR9] Boyle CA et al (2011) Trends in the prevalence of developmental disabilities in US children 1997–2008. Pediatrics 127:1034–1042. 10.1542/peds.2010-2989 10.1542/peds.2010-2989 21606152

[CR10] Caro-Llopis A, Rosello M, Orellana C, Oltra S, Monfort S, Mayo S, Martinez F (2016). De novo mutations in genes of mediator complex causing syndromic intellectual disability: mediatorpathy or transcriptomopathy?. Pediatr Res.

[CR11] Carrera I, Janody F, Leeds N, Duveau F, Treisman JE (2008) Pygopus activates Wingless target gene transcription through the mediator complex subunits Med12 and Med13. Proc Natl Acad Sci USA 105:6644–6649. 10.1073/pnas.0709749105 10.1073/pnas.0709749105 PMC2373359 18451032

[CR12] Chen XF et al (2012) Mediator and SAGA have distinct roles in Pol II preinitiation complex assembly and function. Cell Rep 2:1061–1067. 10.1016/j.celrep.2012.10.019 10.1016/j.celrep.2012.10.019 PMC3513640 23177621

[CR13] Clark RD (2009). FG syndrome, an X-linked multiple congenital anomaly syndrome: the clinical phenotype and an algorithm for diagnostic testing. Genet Med.

[CR14] Conaway RC, Sato S, Tomomori-Sato C, Yao T, Conaway JW (2005). The mammalian Mediator complex and its role in transcriptional regulation. Trends Biochem Sci.

[CR15] Daniels DLF, Schwinn M, Benink MK, Galbraith H, Amunugama MD, Jones R, Allen R, Okazaki D, Yamakawa N, Futaba H, Nagase M, Espinosa T, Urh JM, M. (2013) Mutual Exclusivity of MED12/MED12L, MED13/13L, and CDK8/19 paralogs revealed within the CDK-mediator kinase module. J Proteom Bioinf S2

[CR16] Davis MA, Larimore EA, Fissel BM, Swanger J, Taatjes DJ, Clurman BE (2013). The SCF-Fbw7 ubiquitin ligase degrades MED13 and MED13L and regulates CDK8 module association with. Mediator Genes Dev.

[CR17] de Ligt J (2012). Diagnostic exome sequencing in persons with severe intellectual disability. N Engl J Med.

[CR18] Deciphering Developmental Disorders S (2015) Large-scale discovery of novel genetic causes of developmental disorders. Nature 519:223–228. 10.1038/nature14135 10.1038/nature14135 PMC5955210 25533962

[CR19] Deciphering Developmental Disorders S (2017) Prevalence and architecture of de novo mutations in developmental disorders. Nature 542:433–438. 10.1038/nature21062 10.1038/nature21062 PMC6016744 28135719

[CR20] Dinkel H (2016). ELM 2016—data update and new functionality of the eukaryotic linear motif resource. Nucleic Acids Res.

[CR21] Donnio LM (2017). MED12-related XLID disorders are dose-dependent of immediate early genes (IEGs) expression. Hum Mol Genet.

[CR22] Drozdetskiy A, Cole C, Procter J, Barton GJ (2015). JPred4: a protein secondary structure prediction server. Nucleic Acids Res.

[CR23] Hantsche M, Cramer P (2017). Conserved RNA polymerase II initiation complex structure Curr. Opin Struct Biol.

[CR24] Harms FL et al (2017) Mutations in EBF3 disturb transcriptional profiles and cause intellectual disability, ataxia, and facial dysmorphism Am J Hum Genet 100:117–127. 10.1016/j.ajhg.2016.11.012 10.1016/j.ajhg.2016.11.012 PMC5223027 28017373

[CR25] Ikai A (1980). Thermostability and aliphatic index of globular proteins. J Biochem.

[CR26] Iossifov I (2014). The contribution of de novo coding mutations to autism spectrum disorder. Nature.

[CR27] Kernohan KD et al. (2017) Matchmaking facilitates the diagnosis of an autosomal-recessive mitochondrial disease caused by biallelic mutation of the tRNA isopentenyltransferase (TRIT1) gene. Hum Mut 38:511–516 10.1002/humu.23196 10.1002/humu.23196 28185376

[CR28] Kircher M, Witten DM, Jain P, O’, Roak BJ, Cooper GM, Shendure J (2014). A general framework for estimating the relative pathogenicity of human genetic variants. Nat Genet.

[CR29] Knuesel MT, Meyer KD, Bernecky C, Taatjes DJ (2009). The human CDK8 subcomplex is a molecular switch that controls Mediator coactivator function. Genes Dev.

[CR30] Lek M (2016). Analysis of protein-coding genetic variation in 60,706. Hum Nat.

[CR31] Lelieveld SH et al (2017) Spatial clustering of de novo missense mutations identifies candidate neurodevelopmental disorder-associated genes. Am J Hum Genet 101:478–484. 10.1016/j.ajhg.2017.08.004 10.1016/j.ajhg.2017.08.004 PMC5591029 28867141

[CR32] Lyons MJ (1993) MED12-Related Disorders. In: Adam MP, Ardinger HH, Pagon RA, Wallace SE, Bean LJH, Stephens K, Amemiya A (eds) GeneReviews((R)). Seattle (WA) 20301719

[CR33] Malik S, Roeder RG (2005). Dynamic regulation of pol II transcription by the mammalian Mediator complex. Trends Biochem Sci.

[CR34] Martinez F, Caro-Llopis A, Rosello M, Oltra S, Mayo S, Monfort S, Orellana C (2017). High diagnostic yield of syndromic intellectual disability by targeted next-generation sequencing. J Med Genet.

[CR35] Mukhopadhyay A (2010). CDK19 is disrupted in a female patient with bilateral congenital retinal folds, microcephaly and mild mental retardation. Hum Genet.

[CR36] Muncke N (2003). Missense mutations and gene interruption in PROSIT240, a novel TRAP240-like gene, in patients with congenital heart defect (transposition of the great arteries. Circulation.

[CR37] Neveling K (2013). A post-hoc comparison of the utility of sanger sequencing and exome sequencing for the diagnosis of heterogeneous diseases. Hum Mut.

[CR38] Petrovski S, Wang Q, Heinzen EL, Allen AS, Goldstein DB (2013). Genic intolerance to functional variation and the interpretation of personal genomes. PLoS Genet.

[CR39] Poss ZC, Ebmeier CC, Taatjes DJ (2013). The Mediator complex and transcription regulation. Crit Rev Biochem Mol Biol.

[CR40] Prokop JW, Lazar J, Crapitto G, Smith DC, Worthey EA, Jacob HJ (2017). Molecular modeling in the age of clinical genomics, the enterprise of the next generation. J Mol Model.

[CR41] R Core Team (2013) R: A language and environment for statistical computing http://www.r-project.org. Vienna A, R Foundation for Statistical Computing

[CR42] Risheg H (2007). A recurrent mutation in MED12 leading to R961W causes Opitz–Kaveggia syndrome. Nat Genet.

[CR43] Yuen RK (2017). Whole genome sequencing resource identifies 18 new candidate genes for autism spectrum disorder. Nat Neurosci.

[CR44] Samocha KE (2014). A framework for the interpretation of de novo mutation in human disease. Nat Genet.

[CR45] Schwartz CE (2007). The original Lujan syndrome family has a novel missense mutation (p.N1007S) in the MED12 gene. J Med Genet.

[CR46] Sobreira N, Schiettecatte F, Valle D, Hamosh A (2015). GeneMatcher: a matching tool for connecting investigators with an interest in the same gene. Human Mutation.

[CR47] Sollis E (2017). Equivalent missense variant in the FOXP2 and FOXP1 transcription factors causes distinct neurodevelopmental disorders. Human mutation.

[CR48] Tanaka AJ et al (2015) Mutations in SPATA5 are associated with microcephaly, intellectual disability, seizures, and hearing loss. Am J Human Genetics 97:457–464. 10.1016/j.ajhg.2015.07.014 10.1016/j.ajhg.2015.07.014 PMC4564988 26299366

[CR49] Tsai KL, Sato S, Tomomori-Sato C, Conaway RC, Conaway JW, Asturias FJ (2013). A conserved mediator-CDK8 kinase module association regulates Mediator-RNA polymerase II interaction. Nat Struct Mol Biol.

[CR50] van Haelst MM, Monroe GR, Duran K, van Binsbergen E, Breur JM, Giltay JC, van Haaften G (2015). Further confirmation of the MED13L haploinsufficiency syndrome. Eur J Hum Genet.

[CR51] Vissers LE, Gilissen C, Veltman JA (2016). Genetic studies in intellectual disability and related disorders. Nat Rev Genet.

[CR52] Vulto-van Silfhout AT (2013). Mutations in MED12 cause X-linked Ohdo syndrome. Am J Human Genetics.

