# Supplementary figures and images for: De novo mutations in MED13, a component of the Mediator complex, are associated with a novel neurodevelopmental disorder
Source: Hum Genet. 2018 May 8;137(5):375–88. doi: 10.1007/s00439-018-1887-y (PMC5973976; doi:10.1007/s00439-018-1887-y)

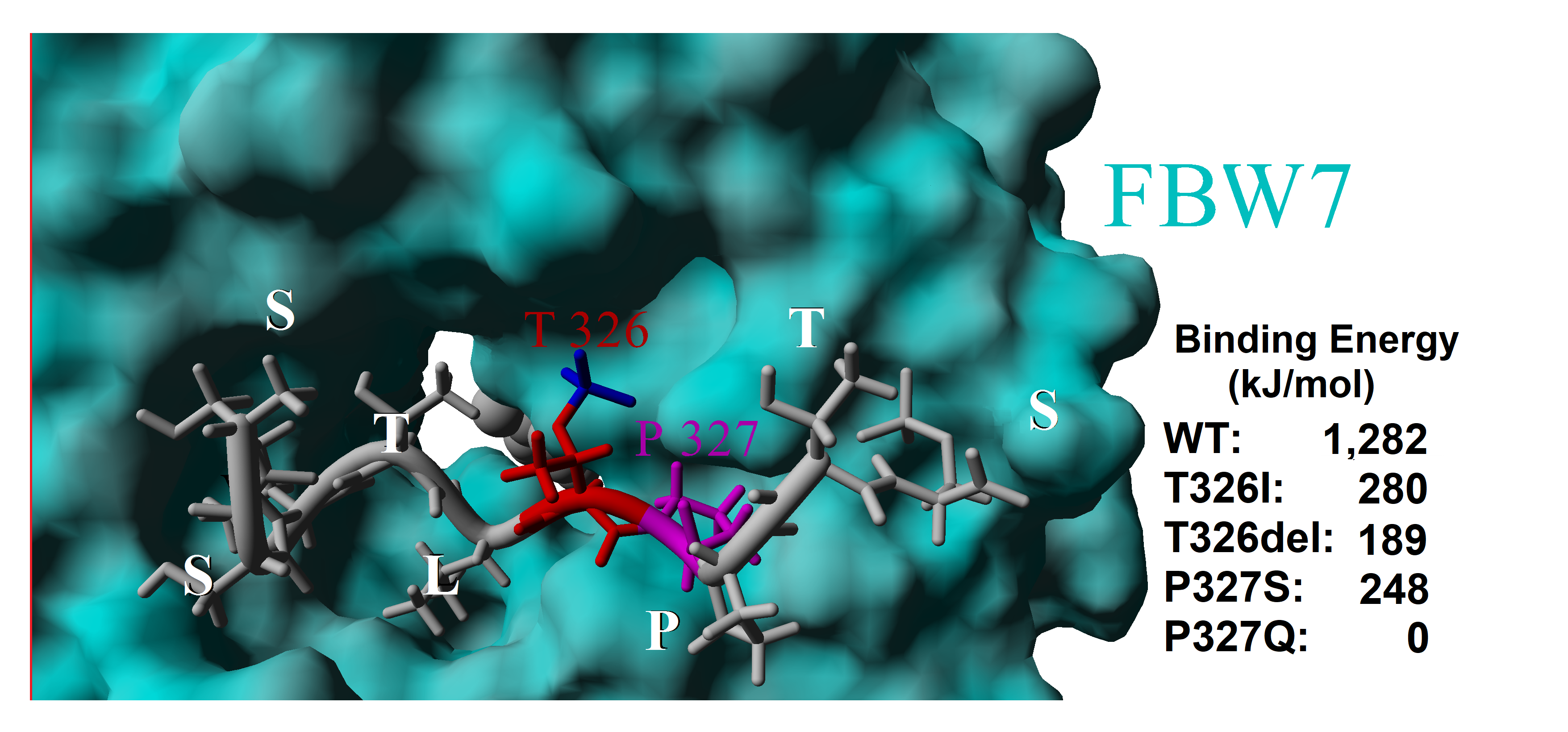

Supplement: Supplementary file 1 — Supplementary Figure 1: Analysis of variants at position 326-327 in relation to Fbw7-interaction. The interaction of MED13 with Fbw7 was modeled, by using PDB structure 2OVQ and amino acids 321-330 of the MED13 protein. All four different variants in our cohort that affect this binding region (T326I, T326del, P327S, P327Q) were subsequently inserted in the model, and binding energy was calculated using AMBER14 force field (http://ambermd.org/) in YASARA. All four variants are predicted to alter the phosphorylation and Fbw7 interaction with a severe decrease in binding energy to Fbw7 (PNG 2012 KB) [file 439_2018_1887_MOESM1_ESM.png]

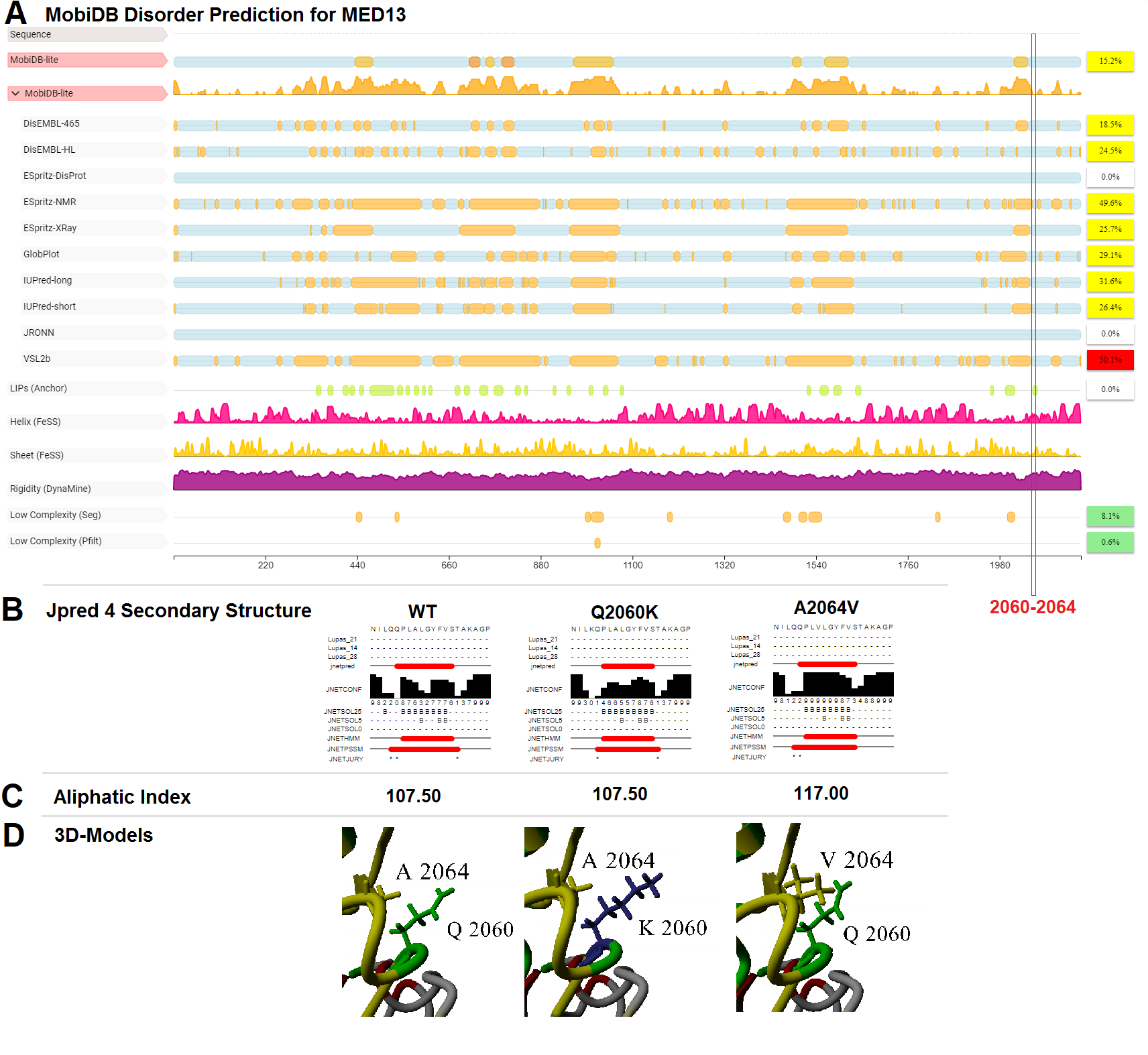

Supplement: Supplementary file 2 — Supplementary Figure 2 Structural packing of MED13 and variants at amino acid position 2060-2064. A) MobiDB breakdown (http://mobidb.bio.unipd.it/Q9UHV7/predictions) for MED13 showing structural disorder (orange) from various databases, linear interacting peptides (LIPs, green) helical prediction (pink), beta sheet prediction (light orange), and rigidity (magenta). The 2060-2064 region is boxed in red with low prediction of disorder and a predicted LIP from 2060-2070, suggesting this highly conserved surface exposed region has a high potential to form secondary structure when bound to some unknown protein binding partner. B) Jpred4 secondary structure predictions(Drozdetskiy et al. 2015) showing predicted changes in secondary structures, with a score of 9 being most likely to form secondary structure at each residue. p.Ala2064Val has highest probability to form stable secondary structure (average residue score of 7.05 compared to the wild type WT 5.6 and p.Gln2060Lys of 5.7). A variant increasing secondary structure would decrease formation rates with the unknown binding partner, thus likely resulting in loss of binding. C) Aliphatic index score(Ikai 1980) showing p.Ala2064Val to increase thermostability of the linear motif. An increase in intrinsic thermostability likely decreases formation rates with the unknown binding partner similar to secondary structure predictions. D) Location and effect of the two missense mutations p.Gln2060Lys and p.Ala2064Val shown on our predicted models for the region (PNG 351 KB) [file 439_2018_1887_MOESM2_ESM.png]
